# Supplementary material for: Are carbon emissions trading and green financial instruments synergistic? -Comprehensive quantitative research based on content analysis
Source: PLoS One. 2024 Mar 7;19(3):e0298601. doi: 10.1371/journal.pone.0298601 (PMC10919601; doi:10.1371/journal.pone.0298601)
Supplement: S1 Data — (ZIP) [file pone.0298601.s001.zip › Data/地级市Z_F工作报告.zip!/║┌┴·╜¡/╦τ╗»╩╨/└┤╫╘╣1⁄2╓┌║┼í╛┬φ┐╦╩2╛▌═°í┐_2015.docx]

各位代表：

我代表市人民政府，向大会报告工作，请予审议，并请市政协委员提出意见。

一、2014年工作回顾

过去一年，在省委、省政府和市委的正确领导下，全面落实市委三届五次全会精神，有力推进“五型经济”、“五个一流”、“八项重点改革”、“十项升级创新”的战略机制措施，主动适应新常态，求真务实，改革创新，全市经济社会平稳发展。实现地区生产总值1295亿元，增长6.7%；规模以上工业增加值246.8亿元，增长8.8%；公共财政预算收入61.5亿元，下降4.5%；社会消费品零售总额462.7亿元，增长13.5%；固定资产投资633.3亿元，增长2.6%；城镇常住人口人均可支配收入19111元，增长11%；农村常住人口人均可支配收入10543元，增长15.1%。

——产业项目和工业经济成效显著。新续建产业项目591个，完成固定资产投资325.2亿元。11个产业园区新增建成面积2.19平方公里，完成基础设施投资11亿元，3个省级工业示范基地晋升为省级产业园区。绿色食品、石化、医药等主导产业带动力、贡献率持续提升，其中绿色食品产业产值、增加值分别占总量的46.5%和38.8%。高新技术产业产值、增加值增幅均超20%。非公经济增加值增长12%，经济总量占全市地区生产总值57%。

——现代农业建设实现新突破。深入推进两大平原现代农业综合配套改革试验，新型经营主体发展到5.2万个，新建现代农机合作社50个，总数达到269个。新增水稻智能催芽车间32处，覆盖水田面积提高24%。完成了国家和省级土地确权试点前期工作。土地流转面积占耕地面积61.2%。建设科技示范带标准化园区572个，104个国家级高产创建示范片获奖励资金3120万元。粮食总产267.8亿斤，实现“十一连增”,绥化市和10个县(市、区)全部进入全国粮食生产先进单位。被确定为“中国绿色蔬菜生产基地北菜南运核心区”。农业综合开发、水土流失治理、植树造林成效明显。新改扩建规模养殖场409个,畜牧业产值、增加值、人均牧业收入同比分别增长12.2%、15.8%和4.6%。

——城乡建设高标准协调推进。房地产投资209.4亿元，建筑面积1354.9万平方米，其中，44个重点集镇完成建筑面积130万平方米，改造泥草(危)房5.33万户。市政设施建设投资25.6亿元。新建改造城市道路70公里98.7万平方米，绿化投入1.76亿元，新建改造公园广场12个，新增绿地126.4公顷。完成既有居住建筑节能改造200万平方米。投入15.3亿元，开复工“三供三治”项目43个。交通重点基础设施投资20.2亿元，改造升级国省道和农村公路1104.5公里，新续建农村客运站30个。

——商贸经济活力进一步增强。大型现代物流企业发展到39家。新增电商、绿色食品旗舰店、品牌连锁店等新型营销主体200多家，分布在全国40多个大中城市，启动建设了绥化绿色食品展示交易中心。新增有进出口经营权企业22家，有进出口实绩企业10家。进出口贸易额3.2亿美元，增长22.5%。实际利用外资2.3亿美元，增长19.7%。金融机构创新农业贷款产品26种，发放涉农贷款412.5亿元。各项存款余额增长14%，贷款余额增长25%。3户企业在“新三板”挂牌上市。

——民生和社会事业保障更加有力。城镇新增就业6.68万人，城镇登记失业率控制在3.69%。企业养老保险新增参保2万人。城乡居民养老保险新增参保5.52万人。在全省率先完成了城乡居民大病保险试点。完成了农村敬老院集中整合。“救急难”工作经验全省推广。开工建设城市棚改15413套，廉租房4562套，公租房1850套。事业单位绩效工资改革深入推进。建设义务教育标准化合格学校22所，各层次培训教师2.3万人次，高标准充实教师队伍753人。投资2.12亿元建设62个县乡医疗卫生机构，投资1.03亿元购置154台套大型医疗设备。县乡公开招聘医护人员875人。投资2.5亿元解决了37.01万农村人口饮水安全。贫困人口减少13%。价调基金“稳价惠民”行动成效明显。文化体育事业健康发展。更加注重生态保护，“黄标车”等节能减排工作完成既定目标。社会治安综合治理、信访、安全生产、食品药品安全、应急管理等工作深入推进，社会和谐稳定。

——政府自身建设全面加强。党的群众路线教育实践活动取得明显成效，突出解决“四风”问题，开展了事关群众切身利益的“十个专项整治”和“六个方面”专项行动，解决群众反响强烈的物业小区管理、建筑工程质量、市容市貌秩序、棚户区改造历史遗留问题等20个方面突出问题。认真办理中央和省委巡视组移交的信访事项，办理情况得到两级巡视组认可。办理人大代表、政协委员建议、提案263件，办结率100%。不断推进依法行政，政府职能进一步转变，行政审批、城市管理改革成效显著，发展环境持续优化。健全完善政务公开机制、制度、程序，政府预算和部门预算全部向社会公开。开展了政府性资金全覆盖审计。市本级完成基建评审项目174个，审减资金3.5亿元。清理规范性文件，行政审批事项减少59.6%，会议下降35.2%，“三公经费”下降10%以上。

——市本级发展迈上新台阶。公共财政预算收入8.9亿元，增长3.3%。绥化经济技术开发区新续建亿元以上产业项目29个，完成投资21.7亿元，税收2.2亿元，增长27%。50件民生实事完成48件，东富村农民新城、黄河小学两件实事因规划和资金问题未能完成。改造建设公园广场5个，新增绿地47公顷，既有居住建筑节能改造10万平方米。改造供水管网5.83公里、新建改造供热管网47.5公里、一水厂升级改造工程投入使用、管道燃气新增入户2.3万户、污水处理二期工程开工建设、城南河污水渠治理进展顺利、生活垃圾焚烧发电项目竣工调试、数字化城市建设基本完成。房地产开发投资17.6亿元，开工建设90万平方米。保障性安居工程开工2607套，首批573套廉租房完成公开分配。

全市公共节能、民族宗教、外事侨务、统计、农业开发、公积金、旅游、残疾人、防震减灾、史志档案、气象人防、老龄双拥等各项工作取得新成绩。

各位代表，这些成绩的取得，是省委省政府、市委正确领导、全市人民团结奋斗的结果。在此，我代表市人民政府，向全市人民，向市人大代表、政协委员，向离退休老领导、老同志，向各民主党派、工商联、人民团体和社会各界人士，向驻绥部队、武警官兵和中省市直各部门，向所有参与、支持和关心绥化发展的朋友们，致以最崇高的敬意和最衷心的感谢!

我们也清醒看到，工作中还存在许多矛盾和问题。一是经济下行压力依然较大，稳增长任务十分繁重，特别是财政收入压力增大、支出扩大，保障能力较弱；二是农业虽大但不强，工业经济总量小，第三产业层次低、发展慢，产业结构不优仍是绥化经济的短板；三是在先天不足、内生乏力的双重考验下，个别新上项目存在低小慢、停产停建问题，扩量提质的任务仍然艰巨；四是广大群众各种利益诉求呈上升趋势，群体访和进京到省非正常访时有发生，加强和改善民生的任务依然较重；五是部分干部适应新常态的思维模式、工作推进方式需要进一步转变,等等。对这些问题，我们将认真加以解决。

二、2015年工作安排

2015年是落实四个全面战略布局的关键之年，是收官“十二五”、谋划“十三五”承上启下的重要一年。要深入落实市委战略要求部署，适应经济发展新常态，紧紧抓住产业项目这个核心，坚持经济工作项目化，持续推进“五型经济”、“五个一流”、“八项重点改革”、“十项升级创新”，紧紧围绕市场导向、政策导向、问题导向，强工、兴农、建城、快牧、活贸、富民，着力提高经济运行质量和效益，着力提高教育卫生等社会事业发展水平，着力改善民生，保持定力、坚定信心，努力实现经济更好更稳更快发展，社会和谐稳定。

主要预期目标是：GDP增长7%以上，规模以上工业增加值增长10%，社会消费品零售总额增长13%，固定资产投资增长10%，公共财政预算收入增长4%，进出口额增长3%，城镇和农村常住人口人均可支配收入分别增长10%。万元GDP综合能耗下降3.2%，氨氮和二氧化硫排放分别下降1.8%和0.2%。

（一）推进产业项目和工业经济加快发展把转方式、调结构放在更加突出的位置，着力培育一批新产品、新业态、新商业模式，增总量壮集群、提质量增效益、扩规模攒后劲、抓管理促规范，推进产业结构不断优化，产业质效不断提升，产业竞争力不断增强。

创新优化招商引资。把扩投资上项目作为实现经济稳增长的根本途径，提高项目质量，省际间到位资金增长10%以上。推进定向招商。围绕本地区独有的优质绿色农牧资源、石化资源等，挖掘放大优势，精选项目，宣传推介，有的放矢。重点突破产业链价值链高端，补强产业链缺失关键环节。农业资源是绥化的最大优势，要从规模现代种植、精深系列加工、市场开拓营销全方位入手，打造成三产融合的全产业链经济、全市最大的优势产业；石化资源是绥化的先天优势，要由原料优势向加工优势、吃配优势转变，由优势传统产业向现代高新产业迈进，形成西部增长极；区位交通资源是绥化的潜力优势，加大第三产业对一二产业的支撑，积极推进传统商贸业向现代服务业转变，培育新的经济增长点。推进重点招商。围绕全市11个产业园区产业定位和功能布局，强化主导产业，完善产业链条,扩大产业规模，增强集聚能力。通过引进增量激活提升原有企业，引进大产业大集团大资本，推进资产重组、联营联合、技术改造，促进园区企业提质增效，做大做强，积极融入产业带、物流带、开放带。推进择优招商。动员全社会力量收集更多的产业、企业、项目信息，科学理性筛选，深入细致论证，围绕提高财税贡献率、投入产出比、就业容量、可持续发展能力，有目的确定招商目标。推进科技招商。突出高新技术成果引进和转化，突出项目的科技领先地位，突出引进成长型科技项目，建立科技企业孵化器，市本级开发区建设科技孵化园，进一步壮大高新技术企业群体。严格执行国家政策、法规，依靠完善基础设施、强化优质服务招商引资。正确处理好项目数量和质量的关系，加强评审，严格把关。不招不适应本地发展的项目，不招对土壤、水、空气有污染的项目，不以牺牲环境为代价去换取一时的经济增长!

突出转方式调结构。投资拉动与创新驱动同步推进，增强内生动力，提高质量效益，提升持续发展能力。促进企业创新发展。大力推进产学研合作，加强创新主体培育，让创新成为驱动发展新引擎。促进产业集群发展。推动产业链延伸、企业裂变，积极发展产业集群，加快绿色食品、石化、医药、纺织、建材等产业向体系完整、功能配套的更高层次迈进。促进园区特色发展。加强道路、电力等基础设施建设，扩展物流、信息集散发布功能，推进优势项目向优势园区集中，提高产业园区的承载力、竞争力和聚集力。完成基础设施建设投资4.5亿元。促进非公经济升级发展。积极发展新材料、新能源等新兴产业，落靠小微企业各项支持政策。非公经济增加值占GDP比重提高1个百分点。

提高全面经营水平。以做大增量带动工业结构调整，向引入要增量，向存量要增量，向高新技术成果转化要增量，向企业改制改组改造要增量，向资源开发要增量，低成本扩张，加速度发展。做活存量。当前提高投产企业生产经营水平、加快在建项目建设速度，是抑制经济下行、拉动经济增长的重中之重，要集中精力、集中力量，抓住不放。扩张数量。持续扩张产业项目和工业经济阵容，新增规模以上工业企业30户。壮大规模。围绕现有经济存量实行数量扩张式、技术升级式、合资合作式发展，推进企业资产重组、联营联合，工业经济在国民经济中的比重稳中有升。提高质量。支持企业对传统设备、工艺进行技术改造和科技创新，推进骨干企业强化支撑，困难企业扭亏为盈。增加效益。引导支持企业切实加强人才队伍建设，完善现代企业制度，强化品牌建设，降低生产成本，增强社会责任，实现企业、财政、社会、生态四个效益协调增长。

提高全程管理能力。继续推进决策咨询进项目、专业服务人员进企业、金融服务进园区和在产业园区建立会计师事务所、律师事务所、资产评估所。更加注重技术改造、产品销售和人才引进，提升服务层次。加强对重点行业和重点企业运行监测，150户重点企业增加值占规上工业比重达到83%，提高8个百分点。

（二）加快现代农业向纵深发展

抓住两大平原现代农业综合配套改革机遇，重点建设绿色农业生态体系，走产加销一体、三产融合现代农业战略发展之路。坚持政府主导、市场运作、发挥优势、区域布局、重点突破、产业拉动、绿色发展、品牌推进，向科技增粮、生态增绿要安全，向新型主体、规模经营要产能，向结构调整、三产融合要竞争力，向金融创新、政策支持要保障，向多元增收、一体化发展要红利，不断转变农业发展方式，推动由农业大市向农业强市迈进。

深化两大平原综合配套改革。各县(市、区)要认真总结三年来农业改革成功经验和存在问题，完善措施，突出抓好“五个创新”，促进生产关系更加适应生产力发展要求。肇东、北林、望奎重点探索新型农业经营主体创新；海伦、兰西、庆安重点探索农村土地管理制度创新；肇东、庆安、望奎重点探索农村金融服务创新；北林、海伦、绥棱重点探索农产品价格形成机制创新；安达、明水、青冈重点探索农业支持政策创新。要高标准完成国家和省改革试点任务，每个县(市、区)结合实际再确定1—2个课题深入研究。规范发展，坚持质量、增加数量，提升各类新型经营主体的自我发展能力，积极推进农民专业合作社联合社建设，建设市级示范社100个。加强农业社会化服务体系建设，为新型经营主体发展提供支撑。创新农村金融服务，结合土地确权等探索融资新渠道，控制好运行风险。各县(市、区)依托金融机构发展村镇银行，推进以土地确权为基础的抵押担保贷款金融产品开发，增加涉农保险内容，农作物保险面积达到1150万亩。积极稳妥引进工商资本进入规模种养、农产品精深加工、农产品流通、新农村建设和农业社会化服务等领域，在更大范围获取资金支持。

积极推进土地流转规模经营。依法规范开展农村土地确权工作。农委负责推进，各县(市、区)全部建立起流转服务平台，市县乡实现网络连接，引导农民加快土地流转，深入探索符合实际的流转模式。发展多种形式的适度规模经营，与城镇化、劳动力转移、生产方式改进、社会化服务水平提高相适应，确保农民得实惠、真受益。

加强科技示范带动。放大整体功能，建设好科技示范园区，提高粮食综合生产能力。坚持由点到线、由线到面、由表及里、表里如一，集现代种植业、规模畜牧业、生态林业、农牧加工业、农田水利化、新型城镇化，多业并举，综合性、多元化、网格式发展。延长示范带、加密园区、增加内容、拓展范围、提高标准，提升示范引带作用，实现提高产量、提高品质、提高卖点、提高效益、降低成本的综合成效。用市场化机制，鼓励支持各类新型经营主体建设示范园区。推进科技集成应用。大力推广浅翻深松、玉米大垄双行密植、水稻智能催芽等农业新技术，全程立标、全程示范。深松整地800万亩，国家级高产创建示范片保持在100个以上。提高机械化水平。提高管理水平，以产业化和产能为纽带，支持农机合作社与专业合作社开展联合合作。推进智能农业与电子商务配套发展，推进农业生产智能化、销售网络化、产品可追溯。各县(市、区)都要积极探索开展智能农业试验示范。

加快绿色农业发展。发挥黑土地自然稀缺优势，加强与市场需求的有效对接，发展绿色农业，做大做强绿色有机食品经济。以市场需求、膳食结构变化调优种植结构、产品结构、经济结构，生产市场适销对路的优质食品，建设优质的工业原料基地和大城市的消费基地。高标准建设生态农业。扩大秸秆还田、测土配方施肥面积。造林绿化14.93万亩。绿色食品认证基地面积提高2.9个百分点。抓住国家加大水利等农业基础设施建设机遇，积极推进阁山水库建设，加大中小河流综合治理力度，除险加固34座中小型水库，发展高效节水灌溉工程45万亩。

大力发展畜牧产业。利用绥化草原和秸秆青贮优势，发展肉蛋奶生产。推进畜牧生产现代化，提高科技示范园区水平。由千家万户散养向规模养殖、养加销一体化转变，鼓励支持集中连片建设标准化规模养殖场、畜牧科技示范园区和现代养殖小区。新建畜牧科技示范园区20个，规模养殖场100个。推进新型养殖经营主体发展。支持畜牧经济合作组织开展畜产品加工、冷链建设、直供直销等业务。推进科技兴牧，抓好原种场、扩繁场建设，抓品种改良，推广和应用先进技术，研究饲草开发利用。强化疫病防控。加强疫情监测检疫，依法监督。

提升农业产业化水平。3000万亩优质耕地是绥化最大、最有作为的宝贵资源，是绿色食品产业的大工厂、加工间，也必将成为全市、全省乃至全国人民的大厨房，是三产融合的大平台，是农民的钱包、企业的钱袋、财政的银行。努力推进从“种的好、养的好”向“加的好、销的好”转变，市场广阔，潜力无限。我们要深入研究从产业端头到田间地头的组织化生产、市场营销，从传统种植向绿色种植转变，从粗放散养向规模化养殖转变，从粗加工向深加工转变，从一般意义现代农业向高端智能农业转变，促进由大基地、大粮仓向大工厂、大厨房、大粮商的产业化升级。要解决好农业量大链短、品牌多而杂、企业小而散等问题。粮食加工转化率达到76%，农产品综合加工能力达到1350万吨。真正把从粮食、蔬菜到秸秆，从种植、加工到营销等各环节利润吃干榨净。

始终抓住农民增收这个核心。增加农民收入是三农工作最最重要的。富裕农民是美丽乡村建设的前提保障、是社会稳定和谐的基石。通过土地流转推进农民进入城镇、工厂、市场等二、三产业，闯进发达地区大门就业创业。增强土地承包物权功能，推进土地由是农民生存保障向成为农民富裕资本转变。积极引导农民加入各种类型的合作经济组织，增加股权资本收入。推进粮经饲三元结构协调发展。支持发展蔬菜、食用菌和林下经济，突出抓好蔬菜保鲜加工、冷链物流配送等前提保障，重点解决“北菜南运”和扩大品牌影响力问题。蔬菜等特色经济作物播种面积占总播种面积的15%以上。

（三）全面提高城乡建设层次品位

坚持政府主导，以规划为先，以需求定建设，以质量促销售，以市场化建设为主体，以功能配套、公益发展为基础，科学合理确定城镇化发展速度、规模和标准，走新型城镇化道路。

积极有序推进城镇村建设。以县城建设为重点，改老城区、建新城区、产业园区产城一体建设。狠抓经济社会发展规划、城乡规划、建设规划、土地利用规划、生态环境保护规划“多规合一”。有序推进节点型中心集镇建设，坚持量力而行、尽力而为，政府重点完善基础设施，市场重点开发建设。坚持撤屯、减村、建镇方向，保有特色，完善村屯布局，政府主导，市场运作，尊重群众意愿，多方式、多形式建设美丽乡村。在乡村建设中，我们要坚持以增加农民收入为核心，使农民搬得起、住得好、富得了。维护好未搬迁村屯的环境和功能，让广大农民住得舒适稳定和谐。

突出投资建设重点。抓住国家实施积极财政政策、扩大内需刺激消费的机遇，积极谋划公路水利等基础设施和医疗卫生教育养老等公共服务项目。完善城市功能。加快城中村、棚户区改造，坚持一水二路三建筑、常年抓植树顺序，加快基础设施升级改造。新城区尽快完善路网、供热、给排水、燃气管网等基础设施。全市“三供三治”工程投资16.3亿元，开复工项目35个。加强公益设施建设。结合城市人口容量和城区空间布局，建设好幼儿园、图书馆、社区医疗、文化娱乐、公共体育等社会事业项目，提高服务设施配套水平。改善人居环境。突出街路绿化、公园广场绿化、小区庭院绿化，全市园林绿化及公园广场建设投资1.78亿元，新建改造公园、广场18个，新增绿地80公顷。加大棚户区改造力度，开工建设1.97万户，配建公租房2335套。

提升城乡管理水平。建设是基础,管理是重点，文化是灵魂。努力实现发展让人民更富裕，文化让绥化更和谐，生态让家园更宜居，城市让生活更美好。抓好城市规划设计的实施，严格执行规划，认真落实设计，强功能、增品位、提质量、树形象。抓好秩序和环境，全面整治市容和交通秩序，突出治理城市“三乱”。创新完善管护机制，定岗定责与标准相结合，提高环境管理标准，推进单位、企业、商铺、住户主体责任制落实。抓好小区物业管理，推进社区与物业管理协调配合，小区物业管理推行市场化，开发企业不能直接管理物业，消除物业管理空白小区。抓好城镇绿化管护，细化落实责任，强化日常管理，完善长效机制，提高管护水平和服务能力。抓好污染治理，建成区内主要环境敏感点区域锅炉全部达标排放。推进农村污染治理，高标准完成165个农村连片整治示范项目建设任务。

（四）加快现代服务业发展

把扩大需求作为经济的新增长点，激发商贸活力，释放消费潜力，让服务业成为就业的新热点、经济发展的新引擎。

积极发展电子商务。加强宣传引导，推动企业顺应信息化发展，深度改变思维理念、商业模式和生产加工、产品营销方式，实现从招商建厂、产品生产到市场营销与电子商务全方位对接。推动电子商务与贸易流通、工业生产、金融服务等相关领域联动发展，降低流通成本，带动实体经济发展，促进就业创业。大力拓展面向“三农”的服务，推动农产品流通，扩大农村就业。建设促进产品营销、居民消费的电子商务平台，支持实体店与电商、线上与线下协同发展。支持各县(市、区)建立绿色食品专业性电子商务平台，进一步拓宽大宗商品交易渠道。积极争取国家农村电子商务示范县项目。

完善城乡市场体系。培育打造区域性批发市场。规范整合现有市场，建设区域性商品交易中心，重点打造一批功能完善、辐射面广的农产品集散中心。积极引进高水平物流企业。市本级重点建设区域性商贸物流中心，发挥好辐射带动作用。规范提升各类专业市场。支持生资、建材、汽车等专业市场建设。加快农产品专业批发市场的标准化、品牌化建设，抓好绿色食品旗舰店和品牌连锁店发展。加强农畜产品仓储物流体系建设。重点引进建设蔬菜保鲜冷库、冷链物流企业，完善仓储配送物流体系。改造完善农贸市场。加快农产品集贸市场退路进场，改造升级经营设施，有条件的向专业批发市场、综合市场转变。

活化创新金融服务。转变思想观念、发展理念，推进融资发展，借力发展，强化资本运营，做好资金变资产、资产变资本文章。加强国有资产运营，推进国有资产资本化。创新城投公司管理体制、运营机制，发挥好投资主体、融资载体、建设实体作用。支持金融机构创新金融产品，鼓励推动企业加大直接融资力度，破解资金制约。支持小额贷款公司、融资性担保公司、政策性担保公司增强实力，加大对小微企业发展支持力度。加强金融监管，优化金融生态环境。

扩大对外开放。积极融入龙江陆海丝绸之路经济带，扩大对外经贸合作领域。推进出口货源基地建设，大力开发符合国际市场需求的绿色、有机和无公害食品，打造绥化出口品牌，扩大地工产品出口规模。依托资源，积极发展特色旅游。

（五）进一步发展民生和社会事业

把民生作为政府工作的重中之重，更加注重保障基本民生，更加注重提高低收入群体生活水平，更加注重养老事业发展，突出群众需求导向，突出问题导向，积极改善民生。

增强社会保障能力。加强养老保险扩面征缴，全市新增2万人。开展被征地农民参加养老保险工作。推进居民大病保险工作。提高社会化养老服务水平，建设公办养老机构3家，新增床位540张。抓好低保提标扩面和清理整顿。提高救助救济保障能力，全面推行“救急难”。进一步健全流浪未成年人救助保护机制，做好儿童救助管理和收养工作。

扩大就业再就业。大众创业、万众创新，多渠道开发就业岗位，继续开展“零就业家庭”就业援助专项活动，加强公共就业服务体系建设。全市新增就业岗位4.7万个，城镇登记失业率控制在4.3%以内。

加快社会事业发展。提高公办幼儿园数量和质量，建设好农村幼儿园，建设义务教育标准化合格学校20所，每个县(市、区)至少办好一所规模较大的职业学校。继续提升高中办学水平，做好教师补充提高。深化医疗卫生体制改革，抓好疾病预防控制工作，加强城乡医保管理，新农合人均筹资达到470元。严厉打击非法行医，维护正常医疗秩序。落实好计划生育政策。加强公共文化体育服务体系建设，完成200处全民健身工程。大力发展公共交通。强化社区建设，提高社区用房标准化水平。

保持社会和谐稳定。把保稳定促和谐放到更加重要的位置。建设好市县乡村四级社会治安综合治理信息平台，抓好基层应急管理示范点。开展重大决策社会风险评估，科学确定评估范围，完善决策审批程序。严厉打击各类违法犯罪活动，提高对动态社会条件下的治安管控能力，更加有效地遏制发案、打击现案，增加群众安全感。深入开展安全生产、食品药品安全隐患排查治理，严防各类安全事故发生。落实领导干部信访工作责任制，推进法制信访、阳光信访，有效解决群众合理诉求。

（六）办好市本级民生实事

全面加快市本级发展，重点抓好经济技术开发区，强力推进招商引资和产业项目建设，促进骨干企业扩产增效、提档升级，努力打造全市产业园区示范区、绿色食品生产样板区、智能农业先行区、统筹城乡发展先导区。重点办好“十项民生实事”，让发展成果更多更公平惠及广大群众。

1、提高养老服务能力。进一步清理规范养老机构，健全完善养老机构健身室、图书室、医务室、娱乐室，对已建成的养老机构具备条件的每处投放5~10万元健身器械。整合加强居家养老、社区养老、机构养老等信息平台建设，实施“养老服务一卡通”工程。支持民办养老机构建设，新增100张床位以上的，给予每床一次性建设补贴4000元。

2、努力增加干部职工收入，提高弱势群体收入水平。按政策规定落实企业职工工资增长机制，确保养老、医保、低保等按政策规定及时足额发放，确保按政策足额兑现工资补贴。继续推进市区低保提标扩面。开展创业培训1500人次，提供小额担保贷款3000万元。开展就业技能培训2000人次。提供妇女创业就业小额担保贷款2000万元。完成厂办大集体改革任务。

3、提高贫困孤残儿童和学生教育救助水平。建设黄河小学。对儿童福利院孤残儿童护理费实施财政专项补助。统筹民政、慈善、工会资金100万元，救助低保家庭贫困大学生200名左右。

4、提高居民医疗水平。完善新农合医疗保险管理。投资3000万元建设绥化市全科医生临床培养基地。

5、加强保障性安居工程建设。实施好投资16.5亿元、7500户棚户区居民的安居工程，配建公租房400套。

6、发展全民健身事业。建设完善大型全民健身活动场所5处，社区健身场地40处。

7、加强街路亮化。在窄旧街路安装节能光伏路灯112盏，启动福和街至广顺街区间街路493盏路灯改造工程，建设开发区兴发路、哈伊路等路灯300基。完成黄河路人行道通行系统和绿化改造。

8、增加绿地广场面积。重点建设碧水、拥军、为民、学苑四处植物园，完成60个住宅小区绿化。在已经建成的植物园、绿地、公园、广场，装备智能音乐系统。

9、加强城市基础设施建设。完成智慧城市“一卡通”建设，城区监控抓拍全程布局。增设公益电子广告宣传栏。启动第二水厂改造，清洗供水管网20公里。积极推进二次供水市场化改造，三年内关闭所有住宅小区生活饮用水水源自备井。改造市区老旧供热管网17.1公里，新增集中供热面积100万平方米。管道燃气新增入户3万户。继续推进武警营房和看守所改造建设。

10、改善人居环境。启动建设污水处理厂和固废垃圾处置二期工程，推进餐厨废弃物资源化利用和无害化处理项目。建设改建住宅楼单元内信报箱1万个。报废黄标车和老旧车辆1500辆。取缔分散供热“小锅炉”27台。进一步完善增加市区公交线路。

三、加强法治政府建设

坚持依宪施政、依法行政，提高法治思维和依法办事能力，切实增强法治意识、规则意识、规矩意识，推进“法治绥化”建设。

为民行政，努力打造履职尽责政府。带头践行社会主义核心价值观，认真践行“三严三实”，巩固群众路线教育实践活动成果。进一步转变作风，深入一线加强调查研究，察实情、解难题。认真落实好上级下放的行政审批事项，强化事中事后监管，推行政府权力清单制度。

依法行政，努力打造民主规范政府。带头尊法学法守法用法。严格重大行政决策法定程序，重要事项集体研究、民主决策。建立完善听证、行政决策风险评估机制，建立重大决策终身责任追究制度和责任倒查机制。严格规范行政执法自由裁量权行使，全面落实行政执法责任制。加大简政放权力度，优化发展环境，规范招商引资行为。

诚信行政，努力打造高效务实政府。率先遵信守诺，完善社会征信体系建设，大力营造诚信环境。政府工作人员坚持坚守正道、弘扬正气，敬畏法律、坚持原则、恪守规矩。强化行政效能监督，严肃惩戒各种效率低下、为官不为行为。

安全行政,努力打造敢于担当政府。统筹落实好生产安全、环境安全、社会安全，加强重点领域和关键岗位风险预警防控。紧紧依靠市委的领导，主动接受人大依法监督、政协民主监督和社会监督、舆论监督，让政府公共权力在监督下运行。

阳光行政,努力打造自律清廉政府。加强财政预算、公共资源配置、重大建设项目、社会公益事业建设等领域的政务信息公开，加快网上审批平台建设。全面落实反腐倡廉各项规定，深入推行“一岗双责”，严肃各项纪律，规范工作秩序，以权力瘦身为廉政强身，坚决惩治腐败!各位代表，人民之愿，政府之责!让我们在市委的正确领导下，以更好的状态、更新的举措、更实的作风，团结一致，奋力拼搏，推进全市经济社会更好更稳更快发展！
